# Gene Structure Evolution of the Na^+^-Ca^2+ ^Exchanger (*NCX*) Family

**DOI:** 10.1186/1471-2148-8-127

**Published:** 2008-04-30

**Authors:** Caly On, Christian R Marshall, Nansheng Chen, Christopher D Moyes, Glen F Tibbits

**Affiliations:** 1 Cardiac Membrane Research Laboratory – Kinesiology, Simon Fraser University, Burnaby, BC, Canada; 2 Department of Molecular Biology and Biochemistry, Simon Fraser University, Burnaby, BC, Canada; 3 Cardiovascular Sciences, Child and Family Research Institute, Vancouver, BC, Canada; 4 Department of Biology, Queen's University, Kingston, ON, Canada; 5 The Centre for Applied Genomics, The Hospital for Sick Children, 101 College Street, Room 14-701, Toronto, ON, Canada

## Abstract

**Background:**

The Na^+^-Ca^2+ ^exchanger (NCX) is an important regulator of cytosolic Ca^2+ ^levels. Many of its structural features are highly conserved across a wide range of species. Invertebrates have a single *NCX *gene, whereas vertebrate species have multiple *NCX *genes as a result of at least two duplication events. To examine the molecular evolution of *NCX *genes and understand the role of duplicated genes in the evolution of the vertebrate *NCX *gene family, we carried out phylogenetic analyses of *NCX *genes and compared *NCX *gene structures from sequenced genomes and individual clones.

**Results:**

A single *NCX *in invertebrates and the protochordate *Ciona*, and the presence of at least four *NCX *genes in the genomes of teleosts, an amphibian, and a reptile suggest that a four member gene family arose in a basal vertebrate. Extensive examination of mammalian and avian genomes and synteny analysis argue that *NCX4 *may be lost in these lineages. Duplicates for *NCX1*, *NCX2*, and *NCX4 *were found in all sequenced teleost genomes. The presence of seven genes encoding *NCX *homologs may provide teleosts with the functional specialization analogous to the alternate splicing strategy seen with the three *NCX *mammalian homologs.

**Conclusion:**

We have demonstrated that *NCX4 *is present in teleost, amphibian and reptilian species but has been secondarily and independently lost in mammals and birds. Comparative studies on conserved vertebrate homologs have provided a possible evolutionary route taken by gene duplicates subfunctionalization by minimizing homolog number.

## Background

The Na^+^-Ca^2+ ^exchanger (NCX) is an integral membrane protein belonging to the Ca^2+^/cation: antiporter (CaCA) superfamily of protein transporters [1]. Catalyzing the reversible counter-transport of three Na^+ ^for one Ca^2+^, NCX plays a major role in maintaining Ca^2+ ^homeostasis in many tissue types [2] although its role is best understood in the heart [3]. In cardiomyocytes, the exchanger extrudes Ca^2+ ^from the cytosol thereby allowing relaxation to occur but it may also function in Ca^2+ ^influx mode under certain physiological conditions, thereby contributing to cardiomyocyte contraction [4]. There is growing body of evidence that the role of NCX in cardiac excitation-contraction coupling differs with developmental and physiological states as well as between species. In the adult mammalian heart, NCX expression levels increase during heart failure and arrhythmias as an intrinsic mechanism of cardiac functional compensation, though with potentially negative side-effects [5]. The expression of NCX in cardiomyocytes is elevated in neonates, suggesting a greater role for the exchanger in early developmental stages [6]. NCX expression also differs among species. For example, NCX expression in teleosts fish cardiomyocytes appears higher than other vertebrates [7]. Interpretation of expression dynamics in physiological, developmental and evolutionary comparisons is complicated by the diversity in *NCX *homologs arising from both splice variants and gene/genome duplications.

NCX is expressed in virtually all tissues across a phylogenetically diverse group of species. Invertebrates appear to express a single *NCX *ortholog based upon analyses in arthropods (fruitfly [8]) and molluscs (squid [9]). The analysis of vertebrate *NCX *homologs has focused mainly on mammals. To date, three *NCX *genes (*NCX1*, *NCX2 *and *NCX3*) have been cloned and functionally characterized from several mammals including mouse [10], rabbit [11], guinea pig [12] and dog [13]. *NCX1 *is expressed ubiquitously [14], whereas *NCX2 *[15] and *NCX3 *[16] are found exclusively in the brain and skeletal muscle. Expression levels generally correlate with the perceived importance of transmembrane Ca^2+ ^flux in a particular cell type (i.e., high in cardiac, neuronal, and kidney tissue but relatively low in liver tissue). An *NCX1 *homolog has been cloned and characterized in several teleosts including trout [17], tilapia [18], and zebrafish [19]. We recently discovered a *NCX *gene (*NCX4*) that has been found only in fish species [20]. Although it has been shown to play a critical role in zebrafish cardiovascular development [21], its functionality and transport phenotype are not known.

The protein products of mammalian *NCX *paralogs range in size from ~800 – 990 residues and show an overall identity of ~66%. Identity is especially high in the N- and C-terminal transmembrane (TM) domains, consisting of 5 and 4 transmembrane segments (TMS), respectively [22,23]. Within each domain, the α-repeats have been suggested to be involved in ion translocation. A large (~550 residues) intracellular loop, which separates the TM domains, contains components important for NCX regulation, such as exchange inactivation by the XIP site and Ca^2+ ^binding by the Ca^2+ ^binding domains (CBDs). The C-terminal portion of the loop of NCX1 is subject to extensive alternative splicing in which differential combinations of six exons (A, B, C, D, E and F) produce five well-documented and abundant splice variants expressed in a tissue-specific fashion, although other variants may be expressed at low levels in certain tissues out of a total of thirty-two possible splice variant exon combinations [24]. The physiological significance of NCX alternative splicing remains controversial. Schulze *et al*. [25] observed distinct voltage dependences between NCX1.1 versus NCX1.3 isoforms whereas Dyck *et al*. [26] and Hurtado *et al*. [27] identified differences between isoforms in Na^+^-dependent inactivation induced by an increase in intracellular Ca^2+^. Therefore a comprehensive evolutionary analysis of NCX alternative splicing is needed.

The origin of many vertebrate gene families can be attributed to whole genome duplications (WGD) early in vertebrate evolution [28]. Superimposed on these genomic events are gene and segmental duplications. The relative importance of these genomic and gene duplications in diversity of *NCX *paralogs is not yet established. Reports of three *NCX *paralogs in mammals are consistent with two rounds of WGD, followed by loss of one paralog. *NCX4 *has been suggested to be a product of gene/genome duplication after teleost divergence from tetrapods [20]. However, the lack of sequence information from a breadth of vertebrates has made it difficult to determine the origin of the vertebrate paralogs. Of particular interest is the origin of the *NCX4 *paralog, which to this point had not been identified in species other than teleosts.

In the current study we build on previous work and present a clearer picture of *NCX *molecular evolution. In addition to protein sequence analysis, closer examination of *NCX *gene structure provides insight into the evolutionary origins of the *NCX *gene family. Since the TMS and specific motifs within the regulatory loop are well conserved among species, examination of the exon-intron boundaries, gene length, intron density, and alternative splice variation provide opportunities for distinctions between possible evolutionary trajectories. Through a combination of existing [20] and newly found *NCX *genomic sequences, a more complete phylogenetic tree can be drawn leading to a new hypothesis of *NCX *gene evolution. These data are consistent with the 1R/2R/3R hypotheses for WGD, generating four *NCX *paralogs in basal vertebrates, and additional paralogs in teleosts. One of these genes, *NCX4*, is present in the genomes of teleost, amphibian and reptilian organisms but has likely been secondarily and independently lost in the mammalian and avian lineages. In addition, certain features of the *NCX *genes have been identified to differ among orthologs and paralogs.

## Results and Discussion

As for most genes, the vast amount of *NCX *genomic data and the speed with which it is expanding far surpasses the availability of cloned and phenotyped *NCX *cDNAs. In this study, therefore, we exploit this by making use of bioinformatics tools to search for *NCX *sequences in all of the genomes currently available including those from the Ensembl browser. Most sequences used in this article are complete but sequences with > 10% of the amino acids missing were not used for phylogenetic analyses. *NCX *searches were performed in genomes from invertebrates and vertebrates, augmented by individual clones from select species (see Table 1).

**Table 1:** Eukaryote NCX Homologs List

Scientific Name	Common Name	NCX
**Mammals**		

*Bos taurus*	cow	1*,2,3*
*Canis familiaris*	dog	1*,2*,3*
*Cavia porcellus*	guinea pig	1*,2*,3*
*Felis catus*	cat	1*
*Homo sapiens*	human	1*,2*,3*
*Macaca mulatta*	macaque	1*,2*,3*
*Monodelphis domestica*	opossum	1*,2,3*
*Mus musculus*	mouse	1,2*,3*
*Ornithorhynchus anatinus*	platypus	1*
*Oryctolagus cuniculus*	rabbit	1*
*Pan troglodytes*	chimpanzee	1*,2*,3*
*Rattus norvegicus*	rat	1*,2*,3*
*Spermophilus tridecemlineatus*	squirrel	1,3

**Aves and Reptiles**		

*Gallus gallus*	chicken	1*,3
*Anolis carolinensis*	green anole	1,2,3,4

**Amphibians and Fish**		

*Xenopus laevis*	clawed frog	1,2,3,4
*Danio rerio*	zebrafish	1a*,1b*,2*a*,2*b*,3,4a*,4*b*
*Gasterosteus aculeatus*	stickleback	1*b*,2*a*,2*b*,3,4*a*,4*b*
*Oncorhynchus mykiss*	rainbow trout	1b*
*Oreochromis mossambicus*	tilapia	1b*
*Oryzias latipes*	medaka	1*a*,1*b*,2*a*,2*b*,3,4*a*,4*b*
*Takifugu rubripes*	fugu	1*a*,1*b*,2*a*,2*b*,3,4*a*,4*b*
*Tetraodon nigroviridis*	green pufferfish	1*a*,1*b*,2*a*,2*b*,3,4*a*,4*b*
*Squalus acanthias*	spiny dogfish	1

**Protochordates and Invertebrates**		

*Ciona intestinalis*	sea squirt	1
*Ciona savignyi*	sea squirt	1
*Aedes aegypti*	mosquito	1
*Anopheles gambiae*	mosquito	1
*Apis mellifera*	honeybee	1*
*Caenorhabditis briggsae*	roundworm	1*,2*
*Caenorhabditis elegans*	roundworm	1*,2*
*Drosophila melanogaster*	fruitfly	1*
*Loligo opalescens*	squid	1*
*Strongylocentrotus purpuratus*	sea urchin	1*

* Represents complete transcripts available in GenBank. **a **and **b **represent teleost NCX duplicates

Gene families in animals arise through gene duplications, but amongst vertebrates the defining events are a series of WGD. Within the chordate lineage, the first WGD (1R) occurred early in vertebrate evolution, around the time of the divergence of the lamprey and hagfish lineages, though the exact timing is not yet clear. A second WGD (2R) occurred prior to the divergence of chondrichthians and osteichthians. Thus, many genes present as single copies in protochordates occur as four copies in chondrichthians and osteichthians, including the sarcopterygian lineage that gave rise to tetrapods. Within the osteichthian lineage, a third WGD (3R) occurred prior to the emergence of teleosts. Thus, an actinopterygian ancestor basal to most teleost fish (including pufferfish, zebrafish, sticklebacks, and medaka) likely possessed four copies of each gene. Additional WGDs occurred more recently in specific vertebrate lineages, such as salmonids [28]. In many cases, gene family sizes in chordates deviate from the predicted 1, 2, 4, or 8 members as a result of duplication or loss of individual genes either before or after WGDs. Based on our analyses, *NCX *gene radiation in chordates is generally consistent with the 1R/2R/3R hypotheses. However, these studies also call into question the conclusions from previous work that hypothesized about the evolutionary origins and phylogenetic distribution of *NCX4 *in fish and tetrapods.

### Invertebrate NCX diversity

Most invertebrate species such as fruitfly, honeybee and mosquito have only one *NCX *gene present in their genomes. A few invertebrate genomes contain several genes with some similarity to *NCX *family motifs, but only one gene found in the genomic sequence appears ancestral to vertebrate *NCX *genes with at least 40% identity to mammalian NCX protein sequences. Multiple *NCX *genes found in invertebrate genomes are most similar to each other with no obvious paralog distinction. *C. elegans *has various putative *NCX *genes but two [GenBank:C10G8.5, Y113G7A.4] have about 45% identity to vertebrate *NCX*. Although in Table 1 we label these gene products as *C. elegans*1 and *C. elegans*2 (according to NCBI annotation), it should be noted that this system does not correspond to our annotation for vertebrate *NCX *paralogs. Other putative *NCX *ESTs (not listed) demonstrated only 15 – 33% protein identity to mammalian NCX sequences. Similarly, in the sea urchin (*Stongylocentrotus purpuratus*), three putative *NCX *genomic sequences were found but only one transcript [GenBank:XM_001184049] displays high identity (~55%) with the vertebrate NCX (others had < 35% identity). Based on the presence/absence of critical motifs, we predict that most of the putative *NCX *genes found in various invertebrate species lack NCX functionality. We conclude that these genes likely arose from lineage-specific duplications after their divergence from chordates because they have: 1) low sequence identity to vertebrate sequences, 2) a lack of conservation in specific amino acids/motifs critical for Na^+^/Ca^2+ ^exchange [3] and 3) weak identity with vertebrate homologs.

### Chordate NCX genes

**Figure 1:**
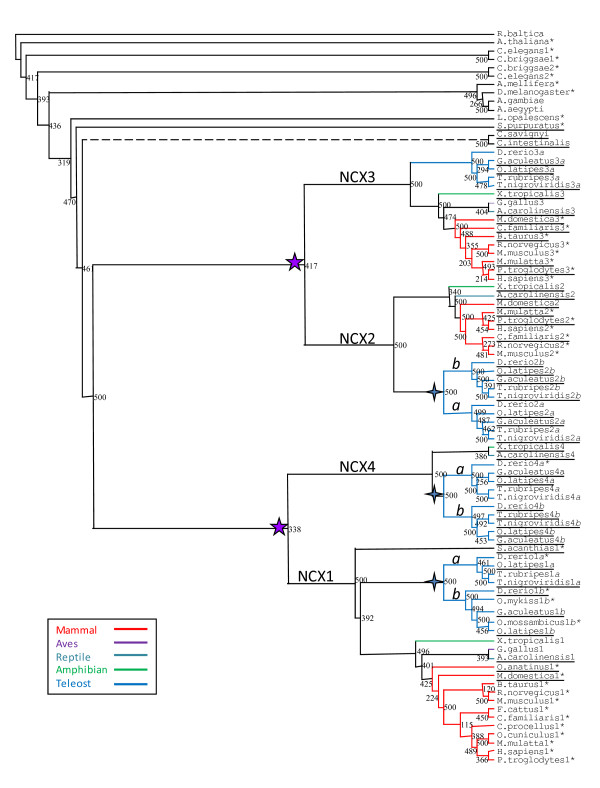
**Maximum Likelihood NCX Phylogenetic Tree**. The ML phylogenetic tree was generated with the PHYML program with 500 replicates using all known and postulated NCX protein sequences. *Pirellula *bacterial strain (*Rhodopirellula baltica*) was used to root this tree. The five-point stars () indicate *NCX *duplication after *Ciona intestinalis *divergence and the four-point stars () indicate teleost-specific *NCX *duplication. Branches are colour coded for vertebrate species, in which red designates mammals, purple indicates birds/aves, teal branches are for reptiles, green are amphibians, and blue are teleosts/fish. The dashed branch (---) indicates the division between invertebrate and vertebrate *NCX *sequences. The asterisk (*) indicates complete transcripts and those without an asterisk are genomic sequences. The letters '*a*' and '*b*' correspond to the teleost duplicate versions of *NCX1*, *NCX2 *and *NCX4*. Underlined species are new *NCX *sequences added in comparison to a previous paper [20].

To better determine the timing of *NCX *duplication events, we searched the protochordate sea squirt (*Ciona intestinalis*) genome for the presence of *NCX *genes. The distinctive evolutionary position of *Ciona *as an invertebrate chordate has been suggested to contain an approximation to the ancestral match of non-duplicated chordate genes; hence it may provide insights into vertebrate evolutionary origins [29]. The *Ciona NCX *genomic sequence is positioned in all of our phylogenetic trees in a manner that provides a clear separation of the invertebrate and vertebrate *NCX *sequences (Figure 1 and see Additional Files 1 and 2). The presence of a single copy of an *NCX *gene in *Ciona *with high percent identity to the various *NCX *genes found in vertebrates is consistent with previous findings that indicate that the *Ciona *genome contains every gene that corresponds to a paralogous family in vertebrates for many signalling molecules, transcription factors and channels [29,30]. The *Ciona NCX *genomic sequence displays ~56% overall identity among vertebrate *NCX *homologs. Even though other genes annotated as *NCX *were found in *Ciona *genome, it is hypothesized that these genes evolved after the divergence of *Ciona *from other vertebrates as evidenced by their lower sequence identity (~33%) to vertebrate *NCX *sequences.

Very little useful *NCX *sequence data are available in cyclostomes (lamprey and hagfish) and chondrichthians. In the lamprey (*Petromyzon marinus*), three partial *NCX *sequences exhibit a high protein sequence identity to NCX paralogs with a wide range (51 – 82%) of identity. One shares high sequence identity with *NCX1*, another with *NCX4 *and the third with both *NCX2 *and *NCX3*. However, the limited data prevent definitive assignment as specific *NCX *orthologs and thus the nature of the cyclostome *NCX *gene family remains unclear. The first extant group to branch from the vertebrate lineage after the 2R WGD is the chondrichthians. Unfortunately, sequence available at this time from the elephant shark (*Callorynchus milli*) was not useful; we found only partial *NCX *genomic sequences with large gaps [31]. However, there is a spiny dogfish (*S. acanthias*) clone that corresponds to *NCX1 *[GenBank:DQ068478].

**Figure 2:**
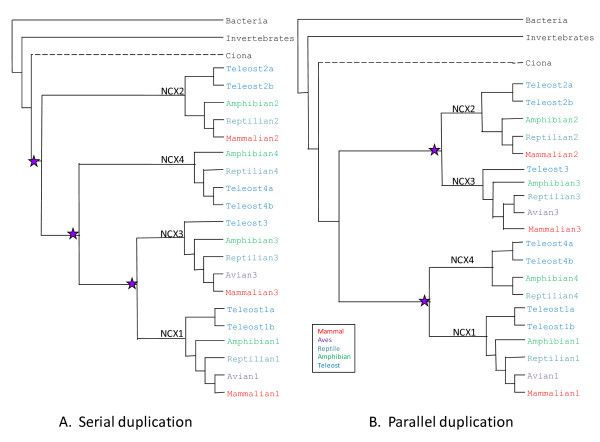
**Summaries of Phylogeny Trees**. Non-scaled, simplified representations of *NCX *evolution, rooted with a bacterial sequence is demonstrated with two manners of duplication: A. serial (Neighbour Joining) and B. parallel (Maximum Parsimony) duplication.

Analysis of *NCX *gene family members in osteichthians and tetrapods is, for the most part, consistent with the 1R/2R/3R hypotheses. The NJ phylogenetic tree shown in Figure 1s supports the notion that the duplication events giving rise to *NCX *paralogs in vertebrates occurred after the divergence of the urochordates, which is estimated to be 520 mya [32]. Therefore, from the existence of one *NCX *gene in invertebrates, duplication events brought about four *NCX *paralogs in basal vertebrates. Hence, the finding of one *NCX *gene in *Ciona's *genome and its positioning in the phylogenetic tree dividing vertebrate from invertebrate branches correlates with previous evolutionary studies [20]. The structures of the Maximum Likelihood (ML) (Figure 1) and Maximum Parsimony (MP) (see Additional File 1) phylogenetic trees indicate two parallel duplications (Figure 2B), which matches the theory of dual genome duplication (2R) which is postulated to have occurred early in the vertebrate lineage [33]. These analyses are consistent with double genome duplication, as observed in the ML/MP phylogenetic trees which show a symmetric topology resulting in four orthologs. However, the phylogenetic tree generated using the Neighbour Joining (NJ) algorithm (see Additional File 2) is consistent with serial *NCX *duplication (Figure 2A). The serial duplication could also be the result of two genome duplications followed by independent gene duplication and loss [34]. However, the bulk of the evidence supports the hypothesis that four *NCX *genes would be expected due to the 2R WGD. Thus, remaining questions concern (1) the anomalous *NCX4 *pattern within tetrapods and (2) the diversification of *NCX *genes in teleosts subsequent to the 3R WGD.

### Evolutionary history of NCX4 genes

Previous studies found a fourth vertebrate NCX (*NCX4*) in genomes of fugu, tetraodon and zebrafish [20]. In zebrafish, the predicted sequence of *NCX4 *on chromosome 7 shows high level of nucleotide identity (> 98%) with two ESTs [GenBank:BQ263135 and BI875890] and a cDNA [GenBank:EF470289[21]]. Likewise, the predicted *NCX4 *sequence in stickleback is identical to an EST encoding the *NCX4 *start coding region up to the XIP site [GenBank:DT993818] and an EST that contains 876 nucleotides from the alternative splice site to the partial last exon [GenBank:DT978254]. There are also two partial sequences from rainbow trout that are similar to fish *NCX4 *orthologs (85–92% identity), including an N-terminal region and a C-terminal comprising 159 amino acids (478 bp) [35]. In this study in which more teleost genomes were available for examination, *NCX4 *orthologs were found in green puffer fish, medaka, and stickleback. Although the best evidence to date is that *NCX4 *occurs widely within teleost fish, its function remains uncertain. Knockdown studies with zebrafish *NCX4 *suggest it is important during development, playing a role in left-right patterning [21].

Previous studies suggested that *NCX4 *occurred in teleosts but not tetrapods [20]. Thus, it appeared most likely that teleost *NCX4 *was a product of either the 3R WGD, or a duplication of *NCX1 *in a basal actinopterygian [20]. When the *X. tropicalis *genome was searched using the Ensembl browser (v31), NCX tBLASTn searches identified only three scaffolds with sequences with the highest identity to NCX. The *NCX *genomic sequences found in scaffolds 684, 572, and 218 were tentatively identified as *NCX1*, *2 *and *3*, respectively. However, a second search in a later Ensembl version (v32) revealed a fourth *NCX *sequence in scaffold 41. We recognized that *NCX1 *is actually located in scaffold 41 while scaffold 684 contains *NCX4 *based on protein sequence alignment (see Additional File 3). Furthermore, the genome of a lizard (*Anolis carolinensis*) also possesses four complete NCX genomic sequences, orthologous to the four sequences found in amphibian and teleosts. The presence of *NCX4 *in these tetrapod genomes argues that *NCX4 *arose during the 2R WGD, rather than in a teleost-specific gene or genome duplication (3R), as previously proposed [20]. With clear evidence of *NCX4 *in basal vertebrates, the apparent absence of the gene in birds and mammals demanded further investigation.

**Figure 3:**
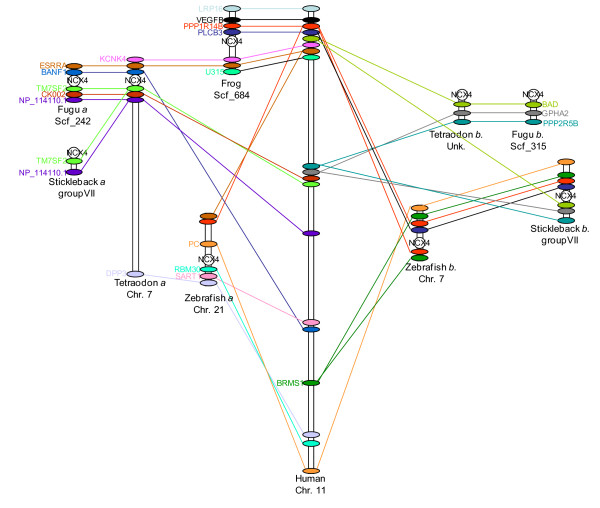
**Synteny Alignment Diagram at *NCX4 *Loci**. Synteny diagram among zebrafish, fugu, frog, tetraodon and human. *NCX4 *(*a *or *b*) gene is represented as a circle and all other surrounding genes are represented as ovals and orthologs are associated with a line.

After extensive searches in all available mammalian genomes for the *NCX4 *gene, it was concluded that this gene is not present. For verification, the Evolutionary Conserved Regions (ECR) Browser [36] was used to find syntenic links among the teleost and amphibian *NCX4 *loci with mammalian counterparts. Syntenic alignments were consistent among the teleost and amphibian *NCX4 *surrounding genes, but high syntenic alignment with the mammalian genomic DNA confirmed that *NCX4 *was completely eliminated in mammals (Figure 3). Mammals arose from the synapsid reptiles very early in reptilian evolutionary history. Thus, the presence of *NCX4 *in a derived reptile (anole) but not mammals suggests that the mammalian homolog was lost in the synapsid reptilian ancestors of mammals, in cynodonts, or in a basal mammal. Note that no evidence of *NCX4 *is found in any mammals studied, including monotremes.

We also failed to find evidence of *NCX4 *orthologs in birds. In our search for *NCX *genes in the available chicken genome (Ensembl v34), only *NCX1 *and *NCX3 *genomic sequences were found in their entirety. Synteny analysis of the complete chicken *NCX *and neighbouring genes confirmed their identity as *NCX1 *(on chromosome 3) and *NCX3 *(on chromosome 5). Partial high identity *NCX *exons were found in three separate unidentified chromosomes. Synteny analysis for the partial chicken *NCX *genes located in an unknown chromosome resulted in alignment with segments corresponding to *NCX2 *genes in other organisms. However, the other partial segments of chicken *NCX *gene resulted in insignificant synteny alignments probably due to the extensive sequence gaps. Therefore, the result is still inconclusive and refinement of the chicken genome will be required to confirm the presence or absence of *NCX4 *in avian species. If *NCX4 *was lost in birds (as in mammals), it must have occurred after the split between lepidosauromorphs (lizards and snakes) and archosauromorphs (crocodiles and bird ancestors). Establishing the exact timing of the *NCX4 *loss would benefit from analysis of a crocodilian genome.

Gene and genome duplication has long been recognized as an important factor in the evolution of the complexity of organismal function and species diversity. The first two vertebrate WGDs provided species with four sets of homologous genes upon which evolutionary processes could act. In many cases, one or more gene became redundant and was/were lost early in lineages, leading to gene families of three or fewer members in tetrapods. The distribution of *NCX4 *in vertebrates is unusual in many respects. First, all four family members were preserved throughout much of vertebrate evolution. NCX is vital in the excitation-contraction coupling in cardiomyocytes. Physiologically, Na^+^/Ca^2+ ^exchangers may differ in electrochemical sensitivity, tissue localization and physiological phenotype. Small differences among each NCX could provide specific needs to different tissues to species that are constantly exposed to fast changing and extreme environments. Thus the preservation of all four family members in most vertebrates would argue that selection acted to preserve the distinct homologs. Thus, the loss of *NCX4 *in mammals, and possibly in birds, might suggest a loss of potential for functional specialization. This is surprising because birds and mammals share the traits of endothermy and high metabolic rate, traits where elevated rates of ion transport occur, particularly in excitable tissues. Further studies of NCX4 expression and tissue specificity would aid in understanding the retention of this gene in teleost, amphibian, and reptile genomes, as well as the independent losses in birds and mammals.

### 3R Whole Genome Duplication (WGD) and teleost *NCX*

**Figure 4:**
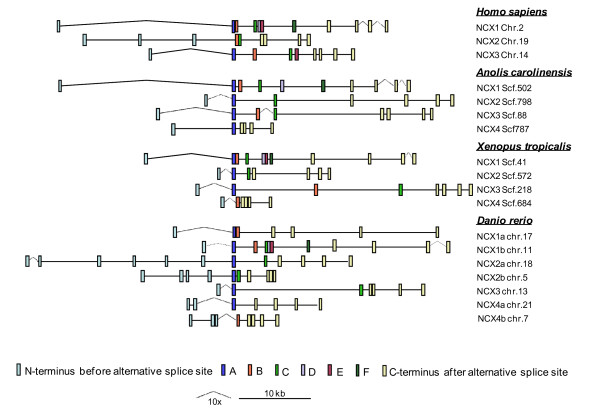
**Sample Species of NCX Homologs Exon Splicing Pattern**. Three sample organisms for mammalian (*Homo sapiens*), reptilian (*Anolis carolinensis*), amphibian (*Xenopus tropicalis*) and teleost (*Danio rerio*) are shown aligned at exon A (blue)/B (orange). Light blue rectangles belong to *NCX *N-terminus before the alternative spliced exons and yellow rectangles represent the C-terminus after the alternative spliced region. Cassette exons (C-F) are indicated in light green, purple, red and dark green where it is applicable and respectively. Distances between exons are scaled as indicated on the legend but not the length of the exons.

**Figure 5:**
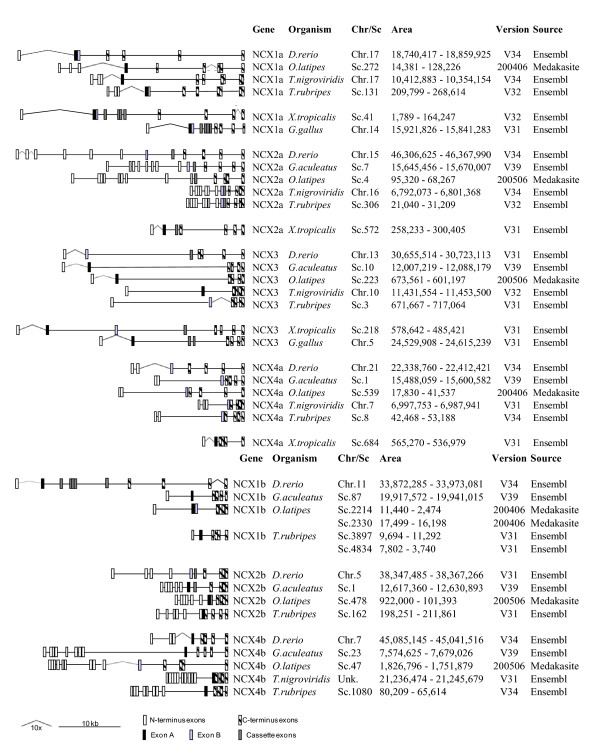
**NCX Gene Homologs and Localization**. Teleost, amphibian and aves list of NCX genes and localization with scaled distances in between exons. Open rectangles represent the exons at the N-terminus located before the alternative spliced exons and the dashed rectangles represent the exons after the alternative spliced exons at the C-terminus. Alternative exons A (black), B (light grey) and the rest of the cassette exons (dark grey) are shown where it is applicable. NCX duplicate genes are separated with NCX*a *and NCX*b *at the top and bottom of the list, respectively.

Superimposed on diversity in *NCX *genes as a result of the 2R WGD is the appearance of additional *NCX *genes in fish, likely as a result of the 3R WGD in teleosts [37]. When we searched for other putative *NCX *paralogs in fish, duplicates were found for all *NCX *genes except for *NCX3*, bringing the number of potential *NCX *genes in teleost species to seven overall (Figure 1, Figure 4 and Figure 5). Each duplicate pair shows high conservation patterns in known TMS, functional regions including α-1 and α-2 repeats, XIP site, Ca^2+ ^binding sites, and exon-intron boundaries.

It is not yet known if the duplicated genes have diverged sufficiently to acquire distinct functions. Langenbacher *et al*. [19] found that the two *NCX1 *genes in the zebrafish demonstrated distinct tissue specificities and effects. They named the *NCX1 *gene duplicates based on their localization in the developing embryo. *NCX1n *was predominantly expressed in the brain and neural tube whereas *NCX1h *was expressed in heart and played a role in its rhythmic contraction. An *NCX1h *knockout causes cardiac fibrillation. In this report, we redefined the distinction between teleost *NCX *duplicate sequences in which "a" is added to the sequences with the highest identity with mammalian NCX homologs and "b" to the most distant. Based upon sequence homologies, *NCX1n *corresponds to *NCX1a*, whereas *NCXh *is *NCX1b*.

Our previous studies on the function of *NCX *homologs focused on trout because of its importance as a model in physiological studies. However, salmonids experienced a lineage-specific WGD [28], and thus there is potential to have duplicates of genes that were duplicated as a result of the 3R WGD. We have cloned and characterized a rainbow trout NCX1 that appears most similar to *NCX1b *of other teleosts (see Figure 1). There are ESTs from the rainbow trout in Genbank that are tentatively identified as *NCX1 *[GenBank: CX255655, CX255654], but the protein sequence has too low identity (71%) to correspond to our NCX1b. However, this EST still has the highest percentage alignment with *NCX1 *genes in comparison to the other three paralogs from other species. It remains to be established if this EST is *NCX1a *or a salmonid duplicated and diverged *NCX1b *variant.

### Organization of NCX genes: central variable segment

In addition to using sequence similarities, we examined *NCX *gene structure (number of exons, intron density range, gene length) to further define the evolution of the *NCX *gene family. All vertebrate *NCX *genes possess similarities in exon structure in both N-terminal and C-terminal regions, and a central segment of the gene that varies more widely among homologs.

In mammalian paralogs, the central variable segment (involved in alternative splicing) of *NCX *genes consists of as many as six exons; *NCX1 *possesses all six exons, whereas *NCX2 *possesses two (BC), and *NCX3 *four (ABCE). Superimposed on the variation in the genetic organization is alternative utilization of the exons in the formation of splice variants, which are thought to be important in conferring tissue-specific functional differences in NCX. When comparing the variable central segment of vertebrate paralogs, all *NCX *genes possess at least one exon that is homologous to either exon A or exon B. Indeed, exons A and B have considerable sequence homology (e.g. a central acidic region), suggesting that they arose through a duplication event early in vertebrate evolution. Based on the sequence similarity (i.e., the maximum likelihood tree), the first WGD event gave rise to the common ancestor of *NCX1 *and *NCX4 *and the common ancestor of *NCX2 *and *NCX3 *(Figure 1). Since both exons (A and B) occur in both mammalian *NCX1 *and *NCX3 *(Table 2), it is likely that the regions were duplicated in a basal vertebrate prior to the 2R WGD. Furthermore, since both exon A and exon B are present in the mammalian, amphibian, reptilian and teleost orthologs of *NCX1*, it argues against a more limited duplication of this segment in selected lineages. In mammals (where it is best studied), splice variants of transcripts possess either exon A or exon B, but not both. The significance of the mutual exclusivity of exon A and B to construct *NCX *is not known, although in general exon A appears in excitable cells such as cardiomyocytes and exon B is expressed in non-excitable cells such as kidney [25].

**Table 2:** General Sequence of Alternative Splice Exons from all NCX Homologs

Species	NCX	A	B	C	D	E	F
Teleost	4all	KLLNVKIIDDEEYEKNKTFTIVLEEPILLEVGQKH					
Amphibian	4	RFIEIQVIDDEEYEKNKNFYVELGEPEMQRSGKKS					
Reptile	4	KYIEIKIIDDEEYEKNKNFHLELGPPVLLDTGPRH					

Teleost	2all		QSFQVRIIDDEEYEKHENFFIVLEEPRWLKRGIS	ALLLNQE			
Amphibian	2		KTLQVKIVDDEEYEKQENFFIILEEPRWMKRGIS	ALLLNQG			
Reptile	2		KSLYVKIIDVEEYEKKDSFFIELGQPRWLKRGIS	ALLLNQD			
Mammals	2		KTLQVKIVDDEEYEKKDNFFIELGQPQWLKRGIS	ALLLNQG			

Teleost	3	KFIHVKIIDDEEYEKNKNFFLELAEPRMVDMSLQK					
Amphibian	3	KTIQIKIFDDEEYEKNKTFFIELREPHLVDLSVQK	KTIRVKIIDEEEYERQESFFIALGEPKWMKRGIS	ALLLNQG			
Reptile	3	KTVHIKVIDDEEYEKNKTFFIELMPPRMVDMSLHK	KTIRVKIVDEEEYERQENFFIALGEPKWMERGIS	AILLNQL			
Aves	3	KTIHIKVIDDEEYEKNKSFFIELMSPRMVDMSLQK					
Mammals	3	KTIHIKVIDDEAYEKNKNYFIEMMGPRMVDMSFQK	KTIRVKIVDEEEYERQENFFIALGEPKWMERGIS	ALLLSPG		GKTSY	

Teleost	1	KTIQINIIDDEEYEKNKNFFLEIGEPQLLEMSERK	KTIKVKILDHEEYDKHANFFIELQEPEWRRRGWT				
Amphibian	1	KIISVKIIDDEEYEKNKTFFLEVGEPRLVEMSEKK	KCITLKILDREEYDKESNFFLVLQEPIWIRRGMK	ALLLNEL	GDFTIT	GKILY	GKPVLRKVQVRDHPIPSTVIILT
Reptile	1	KTISIKVIDDEEYEKNKTFYIEIGEPRLVEMSEKK	KSITLRILDREEYEKECNFYLVLEEPIWIRRRTK	ALLLNEL	GGFTIT		GKPVYRKVHARDHPFPSTVINIQ
Aves	1	KTISIKVIDDEEYEKNKTFYLEIGEPRLVEMSEKK	KFITLRILDREEYEKECSFFLVLGDPVWLRRGVK	ALLLNEL	GGFTIT	GKLWK	GKPVFRKVQARERPLPCTVVTIR
Mammals	1	KTISVKVIDDEEYEKNKTFFLEIGEPRLVEMSEKK	KIITIRIFDREEYEKECSFSLVLEEPKWIRRGMK	ALLLNEL	GGFTIT	GKYLF	GQPVFRKVHAREHPILSTVITIA

**Teleost exceptions**							
Pufferfish	1d	KTIQVNIIDDEEYEKNKNFFIELGDPRLLEMSERK		ARLLQEV			GRDMYRKVQEWHPSAAMINIPGM
Medaka	1d	KTIRINIIDDEEYEKNKNFFLEMGEPLLLEMSERK	KTIALRIMDREEYDKKASFCVELQEPFWNSRWTG	AVLLQEV			
Green pufferfish	1d		KSLHIRIVDDGEFGQDKNFLLELGEPRLLDPSQS				DRDVYRKLQGWNLPDAVINITGM
Trout	1d	KSIQINIIDDEEYEKNKNFFLEMGEPQLLEMSERK		AVLLQEI	GGFVKT		GRDVYRKVQGRDNPVPATIISLA
Tilapia	1d	KTIRINIIDDEEYEKNKNFFLEIGEPRLLEMSERK		AVLLQEV	GGFVKT		GRDIYRKVQGREHPVPSNIISIA
Dogfish	1	KTIEIKVIDDEEYEKNKNFFIEIGEPRLVEMSEKK		ALLLNEL	GPFTKT	AKYFN	GHAIYRKVHFRDNPIPSTVICIA
Zebrafish	1d	KTIQINIIDDEEYEKNKNFFLEIGEPQLVEMSERK		AMLLHEC	GGFVKT	DKQLY	GRDVYRKVQGRDKPIPSTIISIS
Stickleback	1d	KTIQINIIDDEEYEKNKNFFLEIGEPQLLEMSERK	KTIAVRVIDRDEYDKQASFYIELQEPYRNQRRWT	AVLLQEV	GGFVKT		GRDVYRKVQGRDHPAPSAVISIT

The phylogenetic distribution of the remaining exons (C, D, E, and F) is much more variable, though there is a general conservation in the number of exons in the central variable segment of *NCX *orthologs (Table 2). For example, *NCX4 *from teleosts, amphibians, and reptiles possess only exon A; *NCX2 *from fish, amphibians, reptiles, and mammals possess only exons B and C. The exon collection for *NCX3 *orthologs is more variable, with a single exon (A) in teleosts and birds, and additional exons in amphibians and reptiles (ABC) and mammals (ABCE). Exons D and F are present only in *NCX1 *orthologs, but they occur in all vertebrates, suggesting these exons arose in the ancestral *NCX1 *gene shortly after the appearance of the four member gene family.

In general, alternative splicing of exons is an important mechanism to create variation from a single gene. Alternative splicing of exons in the central variable region of *NCX *genes is employed to different extents among paralogs and orthologs. No splice variants are possible for *NCX4 *where only a single alternative splicing exon occurs (Table 2). There are only two exons in this region of *NCX2 *(BC) and no splice variants have been detected in mammals [24]. In the case of *NCX3*, teleosts possess only a single alternative splicing exon, but mammals possess 4 exons (ABDE) and demonstrate multiple splice variants [24]. There is some evidence of alternative splicing for teleost variants of *NCX1*. For example, a cloned rainbow trout *NCX1b *cDNA identified as *TR-NCX1.0 *[17] possessed sequence corresponding to exons ACDF, but a second clone (*TR-NCX1.1*) also contained exon E (unpublished). While it remains possible that alternatively spliced variants of teleost *NCX1 *have simply escaped detection, it appears that splice variants of *NCX1 *are uncommon in fish. This is in contrast to the situation that appears to be seen in mammals, in which splice variants of *NCX1 *are common [24]. Thus, in the case of both *NCX1 *and *NCX3*, splice variants are much more common in mammals than in fish homologs. In the case of *NCX1*, this difference may be related to the duplicate copies of NCX 1, however in the case of *NCX3*, fish did not retain the duplicated *NCX3 *gene [38].

### Organization of NCX genes: N-terminus

Outside of the central variable segment, vertebrate *NCX *paralogs are more highly conserved. It has been reported that exon boundaries of *NCX1 *and *NCX3 *are identical, each with 5 exons (1 N-terminal, 4 C-terminal) [39]. When comparing across vertebrates, the organization of the C-terminal exons is highly conserved, with 4 exons in all *NCX *genes. In contrast, the N-terminus is more variable, and potentially useful for assessing the evolution of the *NCX *family. Amongst the tetrapods, the predominate pattern is a single N-terminal exon. This is seen in *NCX1 *and *NCX3 *of mammals (though *NCX2 *has 3 exons), and all four *NCX *genes in amphibians (Figure 4). In contrast to the conservation seen in tetrapods, the pattern seen in teleosts in much more variable. Although the N terminus of *NCX1b *and *NCX3 *possess a single exon, *NCX1a *and *NCX4a *have 1–3 exons, *NCX2a *has 4 to 8 exons, and *NCX4b *has 3 to 10 exons. *Danio NCX *homologs generally have fewer exons in the N-terminus than do the other teleost *NCX *sequences (Figure 4 and Figure 5).

Based on these limited analyses, it is difficult to determine the factors that drive the variation in N-terminal gene structure in fish. The conservation seen in the gene organization of *NCX3 *is unusual, with a ubiquitous single N-terminal exon seen across fish, or for that matter, across vertebrates. It is also the only member of the *NCX *gene family that lacks a duplicate version in fish. In contrast, the three members that were duplicated in fish each demonstrate variation among orthologs and duplicates. Thus, it appears that the 3R duplication of ancestral genes (each with single N-terminal exons) created a tolerance for genetic rearrangements in this region of the *NCX *genes in the resultant duplicates.

### Organization of NCX genes: gene lengths and intron density

Without accounting for the 5' and 3' untranslated regions (which are mostly unknown), mammalian and reptilian *NCX1 *and *NCX3 *genes average 290 and 135 kb in length in their genomes, respectively, and represent the longest *NCX *genes across species while mammalian *NCX2 *average 30 kb. However, the teleost, amphibian and reptilian genes exhibit the same length patterns but on a smaller scale as expected due to their smaller genome size in comparison to mammalian genomes. A notable exception to this is that of the lizard with a ~94 kb long *NCX2*. The teleost and amphibian *NCX2 *gene duplicates, which are not much smaller than the mammalian homolog, average 22 kb close to the *NCX4a *gene and *NCX4b *at 30 kb. However the avian, amphibian and teleost *NCX1 *and *NCX3 *are greatly reduced in length in comparison to mammalian versions averaging 103 and 65 kb, respectively. Only *NCX1b *demonstrates a shorter gene length pattern in comparison to *NCX1a *across teleost species with an average of 10 kb. Invertebrate *NCX *gene lengths ranged from 7 to 90 kb.

To quantify the evolution of introns, the intron density equation (number of introns per kb of coding sequence) was used to measure the number of introns per *NCX *gene [40]. As shown in Table 3, all teleost *NCX2 *exhibit 2 – 4 introns per kb and *NCX4 *has 2 – 5 introns per kb and all species demonstrate a wide range in intron density. Meanwhile, intron density among all mammals, bird, amphibian, reptile and zebrafish *NCX1a *maintain a consistent value of 1 intron per kb and the rest of the teleost *NCX1a *intron density is consistent at 2 – 3 introns per kb. Also, *NCX3 *gene intron density is preserved among all species at 1 intron per kb. As for the frog, lizard and chicken, all known *NCX *genes within these species contain an average of 1 intron per kb.

**Table 3:** NCX Intron Density

Organism	NCX1a	NCX1b	NCX2a	NCX2b	NCX3	NCX4a	NCX4b
*H. sapiens*	1.32		2.17		1.38		
*C. familiaris*	1.36		2.67		1.38		
*M. musculus*	1.33		2.53		1.38		
*R. novergicus*	1.33		2.17		1.38		
*G. gallus*	1.30				1.44		
*A. carolinensis*	1.44		1.40		1.37	1.00	
*X. tropicalis*	1.33		1.56		1.39	1.48	
*D. rerio*	1.36		3.10	3.14	1.42	1.77	2.16
*T. nigroviiridis*	2.82	1.66	3.42		1.45	2.15	4.66
*T. rubripes*	2.72	1.55	3.86	3.32	1.45	1.81	4.76
*O. latipes*	2.84	1.40	3.75	3.35	1.45	1.76	4.43
G. aculaeatus		1.45	4.12	3.33	1.45	1.76	4.35

* Units = introns per kb of coding sequence (10^-6^)

(a) or (b) applicable for teleost only

The results from both *NCX *gene lengths and intron density demonstrate a correlation of similarities of *NCX2 *with *NCX4 *that differentiate them from *NCX1 *and *NCX3*. Exon-intron boundary and intron density high variations could indicate that *NCX2 *and *NCX4 *are genes with a higher rate of mutation in comparison to *NCX1 *and *NCX3*, which are more conserved and consistent. Gene length comparisons showed more compactness in *NCX2 *and *NCX4*, similar to sea squirt and most invertebrate *NCX *genes. These differences noted in *NCX *gene structure may shed light about their adaptation and evolutionary patterns.

## Conclusion

Rapid growth in the number of sequenced genomes has made it possible to identify a large number of *NCX *genes in many species. Although most NCX expression and function has been characterized from mammalian organisms, *NCX *gene presence and conservation is seen among a great variety of animal sequenced genomes. *NCX *presence in all species' sequenced genomes has allowed the construction of a phylogenetic tree that correlated to animal evolution and revealed that the origin of *NCX *duplication was initiated at the emergence of vertebrate organisms. From this duplication, at least four paralogs are hypothesized to have resulted and these have been found in teleost, amphibian and reptilian genomes, however we postulate that only three endured in mammalian genomes. Also, duplicate forms of these *NCX *genes have been found only in teleost genomes that correlate with the isolated genome duplication in ray-finned fish [38].

The presence of the *NCX4 *gene, found only in teleost, reptile and amphibian genomes, indicates that *NCX *duplicated at least three times after the tunicates (i.e.: *Ciona*) and before the emergence of teleosts. The maintenance of *NCX4 *in teleosts may indicate specific demands significant for efficient and different Na^+^/Ca^2+ ^exchange in organisms with *NCX4*. It has been recently demonstrated that *NCX4 *is expressed in the zebrafish [21] but characterization of its Na^+^/Ca^2+ ^exchange and tissue localization requires exploration. As for the duplicate *NCX *genes in teleosts, only one *NCX1a *from the zebrafish [19] and three *NCX1b *have been cloned and demonstrated to be functional in cardiac tissue in zebrafish [19], trout [17] and tilapia [18]. Zebrafish is the only teleost in which both *NCX1 *have been cloned from cardiac (*NCX1h/NCX1b*) [GenBank:AY934775] and neural tissues (*NCX1n/NCX1a*) [GenBank:AY934776] [19]. The *NCX *gene redundancies in teleosts may indicate subfunctionality and substitute usage of alternative splicing exons as seen in mammalian organisms that have only three *NCX *genes.

When *NCX *sequences are compared among all species, the main areas of conservation (i.e.: TMS and regulatory sites) can be observed. These phylogeny studies can provide an overall evolution of the *NCX *gene family. However, with further gene structure analysis, this report pinpoints the N-terminus segment including the central variable segment to be the main part of the protein to adapt to the requirements of environmental changes as organisms evolve. This is seen in our previous reports [41] in which temperature dependence comparisons between a mammalian and rainbow trout *NCX1 *differ due to sequences differences in the NH_2_-terminal transmembrane segment.

## Materials and Methods

### Data Mining of NCX sequences

tBLASTn searches were performed using known NCX protein sequences in available genome sequences in ENSEMBL database. Predicted and mRNA sequences of interest have yielded over 100 candidate genes for NCXs from GenBank, Ensembl, University of California, Santa Cruz Genome browser, and Elephant Shark genome project website [31]. Species names, gene names and location are listed in Table 1. The available genomic sequences of invertebrate and vertebrate species were analyzed to identify homologous NCX sequences. Due to some inaccuracies and missed annotations of *NCX *genes in Ensembl, the tBLASTn search method was utilized to find/correct predicted exons and complete segmented NCX protein sequences with cloned mammalian NCX protein sequences. Identification of candidate NCX sequences was based on conservation within the 9 TMSs, α-repeats and regulatory sites, originally established by known sequences. Hence, we have been able to categorize and/or complete over 35 NCX protein sequences in 10 different species' genomes.

Targeted databases included: the non-redundant protein and nucleotide databases at NCBI BLAST [42] and the Ensembl Genome Browser v31–v34 and v39 [43] as seen in Table 1. The *NCX *ESTs (expressed sequenced tags) and clones were obtained from NCBI Nucleotide database [44]. The lizard (Feb.2007) sequences were obtained from the University of California Santa Cruz (UCSC) Genome Browser [45].

### Phylogenetic analyses

To understand the origins of the eukaryotic *NCX *family, we performed a phylogenetic study of all *NCX *sequences from all species. Multiple sequence alignments and bootstrapped Neighbour-Joining rooted trees were prepared using ClustalX (v. 1.83) [46]; alignments were manually edited with Genedoc to remove the alternative spliced exons and visualized with TreeView32. Bootstrapping was performed (1000 replicates), and most nodes demonstrated high confidence values. PHYLIP-3.63 (.)[47] was used to construct 1000 Maximum Parsimony trees and PHYML online [48] was used to obtain 500 Maximum Likelihood trees and a consensus tree for each algorithm is shown with TreeView32. Protein sequence names were assigned according to species scientific name and *NCX *homolog number (i.e. = human NCX1 is labelled as H. sapiens1). Incomplete *NCX *sequences missing over 10% of the total amino acids were not included in the phylogenetic tree although some are listed in Table 1. A related *NCX *from the bacterium pirellula (*Rhodopirellula baltica*) was used as the outgroup to root the phylogenetic tree.

### Synteny alignment

The Ensembl database and Evolutionarily Conserved Regions (ECR) browser [49] were used to locate syntenic regions among teleost, avian, and mammalian genomes. In the ECR browser, a scaffold section known to contain the *NCX4 *gene flanked by a minimum of 30,000 base pairs was used to align with all other available genomes to find synteny patterns. The Ensembl database was used to find the predicted orthologs in the genomic regions found in the ECR browser.

## List of Abbreviations

Sodium-Calcium exchanger: NCX; Chromosome: Chr; Scaffold: Scf; duplicate: dupl./d; contig: cntg; unknown: unk; basepairs: bp; kilobasepairs: kb.

## Supplementary Material

Additional file 1 **Figure S1. Maximum Parsimony Phylogenetic Tree**. A consensus Maximum Parsimony tree that was obtained with 1000 replicates from the Phylip program. Click here for file

Additional file 2 **Figure S2. Neighbour-Joining Phylogenetic Tree**. A Neighbour-Joining phylogenetic tree that was obtained with 1000 replicates from the ClustalX program. Click here for file

Additional file 3 **Figure S3. Teleost, amphibian and reptilian NCX4 amino acid alignments**. NCX4 transcript and genomic sequences found in teleost, amphibian and reptilian genomic data have been aligned with ClustalX and viewed with Genedoc. Click here for file
