# Supplementary material for: Gene Structure Evolution of the Na+-Ca2+ Exchanger (NCX) Family
Source: BMC Evol Biol. 2008 Apr 30;8:127. doi: 10.1186/1471-2148-8-127 (PMC2408596; doi:10.1186/1471-2148-8-127)
Supplement: Additional file 1 — Figure S1. Maximum Parsimony Phylogenetic Tree. A consensus Maximum Parsimony tree that was obtained with 1000 replicates from the Phylip program. [file 1471-2148-8-127-S1.doc]

**Figure S1 Maximum Parsimony Phylogenetic Tree**

A consensus Maximum Parsimony tree was obtained with 1000 replicates from the Phylip program. The species labelling and branch colouring are the same as the previous tree.
