# Supplementary material for: Gene Structure Evolution of the Na+-Ca2+ Exchanger (NCX) Family
Source: BMC Evol Biol. 2008 Apr 30;8:127. doi: 10.1186/1471-2148-8-127 (PMC2408596; doi:10.1186/1471-2148-8-127)
Supplement: Additional file 3 — Figure S3. Teleost, amphibian and reptilian NCX4 amino acid alignments. NCX4 transcript and genomic sequences found in teleost, amphibian and reptilian genomic data have been aligned with ClustalX and viewed with Genedoc. [file 1471-2148-8-127-S3.doc]

**Figure S3. Teleost, amphibian and reptilian NCX4 protein alignments**

NCX4 transcript and genomic sequences found in teleost, amphibian and reptilian genomic data have been aligned with ClustalX and viewed with Genedoc. All sequences were extracted from genomic data except for the zebrafish NCX4 (D.rer4) which is a transcript from GenBank (NM_001089419). The black shading indicates 100% conservation among the sequences listed, grey shading in white letters has at least 80%, grey shading in black letters has 60% and no shading indicates less than 60% in conservation. The dashes (-) indicate gaps. Shorten names were used in the alignment for the green pufferfish (T.nig), fugu (T.rub), medaka (O.lat), stickleback (G.acu), lizard (A.car) and Western clawed frog (X.tro).
